# Effect of the Compositions on the Biocompatibility of New Alumina–Zirconia–Titania Dental Ceramic Composites

**DOI:** 10.3390/ma13061374

**Published:** 2020-03-18

**Authors:** Amani Khaskhoussi, Luigi Calabrese, Monica Currò, Riccardo Ientile, Jamel Bouaziz, Edoardo Proverbio

**Affiliations:** 1 Department of Engineering, University of Messina, Contrada di Dio Sant’Agata, 98166 Messina, Italy; eproverbio@unime.it; 2 National Interuniversity Consortium of Materials Science and Technology, INSTM, Via Giuseppe Giusti 9, 50121 Firenze, Italy; 3 Department of Biomedical and Dental Sciences and Morphofunctional Imaging, University of Messina, Via Consolare Valeria, 98123 Messina, Italy; moncurro@unime.it (M.C.); ientile@unime.it (R.I.); 4 Laboratory of Industrial Chemistry, University of Sfax, National School of Engineering, Sfax 1173-3038, Tunisia; Jamel.bouaziz@gmail.com

**Keywords:** ceramic composites, wettability, genotoxicity, cytotoxicity, dental application

## Abstract

Dental implant biomaterials are expected to be in contact with living tissues, therefore their toxicity and osseointegration ability must be carefully assessed. In the current study, the wettability, cytotoxicity, and genotoxicity of different alumina–zirconia–titania composites were evaluated. The surface wettability determines the biological event cascade in the bioceramic/human living tissues interface. The measured water contact angle indicated that the wettability strongly depends on the ceramic composition. Notwithstanding the contact angle variability, the ceramic surfaces are hydrophilic. The cytotoxicity of human gingival fibroblast cells with materials, evaluated by an (3-(4,5 methylthiazol-2-yl)-2,5-diphenyl-tetrazolium bromide (MTT) test, revealed an absence of any cytotoxic effect. A relationship was found between the cell viability and the wettability. It was subsequently deduced that the cell viability increases when the wettability increases. This effect is more pronounced when the titania content is higher. Finally, a comet test was applied as complementary biocompatibility test to detect any changes in fibroblast cell DNA. The results showed that the DNA damage is intimately related to the TiO_2_ content. Genotoxicity was mainly attributed to ceramic composites containing 10 wt.% TiO_2_. Our research revealed that the newly developed high performance alumina–zirconia–titania ceramic composites contain less than 10 wt.% TiO_2_, and display promising surface properties, making them suitable for dental implantology applications.

## 1. Introduction

One of the most important challenges in the dental implantology is the substitution of metal alloys with more biocompatible materials such as ceramic to overcome the biocompatibility issues [1,2,3,4]. Indeed, dental ceramics have attracted researchers’ attention thanks to their good chemical resistance, excellent mechanical and physical properties, and especially their high biocompatibility. These performances can reduce the negative biological reactions to the implants such as the aseptic loosening and the osteolysis [5].

Alumina, or aluminum oxide (Al_2_O_3_), was the first ceramic used for biomedical applications because of its high hardness and chemical and aesthetic properties. Nevertheless, the modest toughness of alumina is also its main deficiency. Indeed, the high rate of fractures in various alumina-based implants was reported on by clinical evaluations [6]. Zirconia (ZrO_2_) ceramics were then introduced in implantology due to their resistance to fracture, their toughness (which is higher than the alumina), and their excellent biocompatibility [7]. However, zirconia bioceramics have a low resistance to ageing due to their transformation from the tetragonal phase (t) to the monoclinic phase (m) in the human body. This transformation is accompanied by an expansion of about 4% to 5% in volume. This volumetric expansion creates compressive stresses resulting in the drop of mechanical performances [8]. 

Up to now, great efforts have been made to produce a new class of dental biomaterials with high mechanical performances. The combination of the high stiffness of alumina and the excellent toughness of zirconia could be an effective strategy [9]. Nevertheless, the previous works on alumina–zirconia ceramic composites revealed complex and high cost manufacturing processes such as hot-press sintering [10].

Furthermore, several additives, such as TiO_2_, have been used in order to improve the sintering and enhance the ceramic performances of composites [11]. TiO_2_ has also showed excellent bioactivity, promoting the attachment of implants with living bone tissues in a short time [12]. Al_2_O_3_-ZrO_2_-TiO_2_ ceramic composites for dental application have recently been developed [13,14,15]. The composition, physical properties, microstructure, and processing techniques of these new ceramic composites are different from those of the conventional ones used in dentistry, thus potentially influencing their inertness. Thus, biosafety cannot be deduced from the analysis of one ceramic composition to other formulations or conditions [16]. 

In our previous works [13,14,15,17], we successfully elaborated and optimized ternary Al_2_O_3_-ZrO_2_-TiO_2_ ceramic composites, through an easy and low-cost manufacturing process, with high mechanical performances (stiffness, strength, and toughness) and an exceptional aging resistance. Indeed, our fabricated ternary ceramics showed higher aging resistance than the commercial zirconia based dental ceramics without any significant degradation of mechanical performances over a simulated duration of 40 years of clinical use [18].

Based on our promising results, an improvement in the knowledge of the biocompatibility of these ternary ceramic composites is required in order to consider them potentially effective for dental implantology applications. 

The aim of this work is to study how the surface behavior (wettability) can influence the cytotoxicity and the genotoxicity of the ternary mixtures of Al_2_O_3_-ZrO_2_-TiO_2_ ceramic composites with the purpose of identifying the key factors in determining the optimal formulation for dental implantology. The surface wettability of materials was studied through the static contact angle. In vitro studies were conducted on human gingival fibroblast (HGF) cells. An MTT (3-(4,5 methylthiazol-2-yl)-2,5-diphenyl-tetrazolium bromide) test was used for the determination of cell proliferation. DNA damage was evaluated using a comet assay.

## 2. Materials and Methods 

### 2.1. Ceramic Composites Manufacturing

Al_2_O_3_ (<50 nm), TiO_2_ (<21 nm), ZrO_2_ (12% CeO_2_) powders with purity ≥99.5%, purchased from Aldrich, were utilized for composite ceramics preparation. After carefully weighing, powders with absolute ethanol were homogeneously mixed and then dried at 80 °C. Afterwards, the mixtures of different powders were molded by uniaxial cold pressing (150 MPa) to obtain green pellets (diameter = 10 mm, thickness = 3 mm). All pellets were sintered for 2 h in a digital furnace in air at 1400 °C followed by controlled furnace cooling. All composites underwent the same preparation steps and have a comparable roughness (Ra < 0.5 μm).

Table 1 summarizes the details of the different prepared specimens. All specimens were coded by TxAxZx, X represents the amount (wt.%) of each added component.

Microstructural and mechanical characterization and aging stability of ceramic mixtures have been reported in previous works [13,14,15].

### 2.2. Wettability Measurements

The bone cells are anchorage cells and have been shown to adhere better on material with high wettability [19,20,21]. Thus, the control of the wettability is an important requirement that has to be fulfilled in order to obtain a bioceramic material with good biological performance, and is an interdisciplinary challenge. For this reason, the surface wettability of the sintered ceramic composite was studied through the static contact angle technique using a sessile drop measurement machine (optical tensiometer Attension by Biolin Scientific, Gothenburg, Sweden). A 2 μL droplet of bidistilled water was set on the composite surface in atmospheric condition at 25 °C. The drop image was taken by a micro CCD (Charge-Coupled Device) camera and automatically analyzed by a suitable Attension software (OneAttension by Biolin Scientific, Gothenburg, Sweden) with a drop profile fitting (Young–Laplace) in order to measure the contact angle (CA) of the bidistilled water droplet on the ceramic surface. Five replicas for each measurement were carried out.

### 2.3. Cell Culture and Treatment

HGF-1 cells, human gingival fibroblast (ATCC-CRL-2014), were cultured in complete D-MEM (Dulbecco’s modified Eagle’s medium) supplemented with 10% fetal bovine serum supplemented with penicillin 100 U/mL and streptomycin 100 μg/mL. All these products were purchased from Sigma-Aldrich (St. Louis, Missouri, United States). HGF-1 cells were then plated at a density of 2 × 10^5^ cells/mL in plates and chamber slides containing the discs and incubated for 3 days at 37 °C in an incubator with 5% CO_2_. 

### 2.4. Cell Viability Assay

The mitochondrial activity of living cells was evaluated via the quantitative colorimetric MTT assay to understand the effect of the formulation of different ceramics on the human gingival cells’ viability.

After incubation, HGF-1 cells were treated at 37 °C for 4 h with MTT (concentration = 0.5 mg/mL). Afterwards, the mitochondrial dehydrogenases in viable cells produced insoluble purple formazan crystals. The acidic solution 0.04 M HCl in absolute isopropanol was used to dissolve these crystals at 37 °C for 4 h. Then, the microplate reader (Sunrise, Tecan, Männedorf, Switzerland) was used to evaluate the optical density at 570 nm and the cell viability percentage was calculated by the absorbance ratio of exposed cells vs control cells.

### 2.5. Analysis of Cell Morphology

At the end of treatment, HGF-1 cells were fixed with methanol at −20 °C for 20 min, and permeabilized with 0.2% TritonX-100 in PBS (phosphate-buffered saline). Then, specimens were incubated with Turk’s reagent for 3 min at room temperature, and washed with PBS. A Leica DM IRB microscope (Laborlux K, Leica Microsystems GmbH, Heidelberg, Germany), equipped with Canon Power Shot S50 camera (Canon, Ohta-ku, Tokyo, Japan), was used to analyze the morphology of the stained specimens.

### 2.6. Single-Cell Gel Electrophoresis (SCGE, Comet Assay)

The DNA damage was assessed using the SCGE method, also known as comet assay, as previously described [22]. The fibroblast cells were briefly harvested from suspension and then embedded in a thin layer of agarose gel (1% low melting) upon clear microscope slides. Cells were lysed and DNA was unwound under alkaline conditions (1 mM EDTA and 300 mM NaOH–pH 13) for 20 min. Afterwards, electrophoresis was employed for 30 min at 300 mA and 25 V (*0.86 V/cm). Following electrophoresis, the slides were washed with 0.4 M tris(hydroxymethyl)aminomethane (Tris) (pH 7.5) and stained with ethidium bromide (2 µg/mL). Analysis was carried out by using a DM IRB fluorescence microscope (Leica Microsystem, Heidelberg, Mannheim, Germany), equipped with a digital recording Power Shot S50 camera (Canon, Milan, Italy). Two replicas for each batch were carried out. Images of 50 cells per slide (100 cells per batch) were randomly acquired and analyzed using CASP software (v.1.2.3, Casplab, Wrocław, Poland). The featured parameters were: length (TL), percentage of DNA (% T-DNA) and moment (TM) of the tail. All values were statically evaluated by one-way ANOVA (Analysis of Variance).

## 3. Results and Discussion

### 3.1. Wettability 

**Figure 1:**
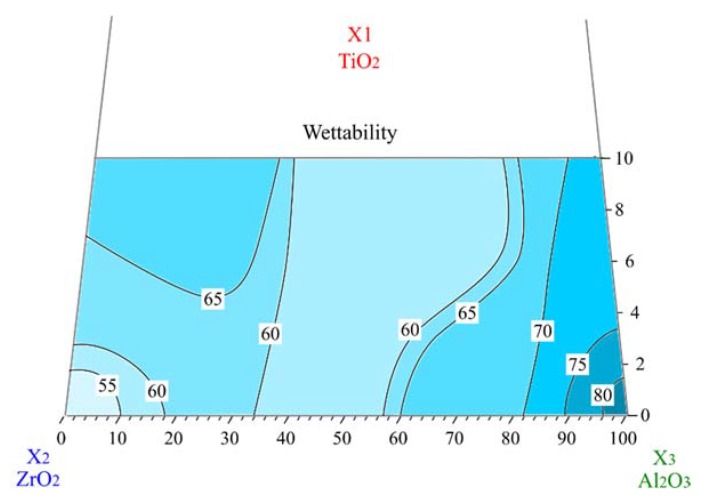
Contact angles of different ceramics with water on a ternary TiO_2_-Al_2_O_3_-ZrO_2_ plot.

The results of the contact angle measurement used to evaluate the wettability of the different ceramic composites are illustrated in Figure 1. The contact angle is a quantitative measure of the wetting behavior of the solid by the liquid. The interaction between the solid surface and the liquid surface is, in fact, strongly related to surface energy. High surface energy generally promotes high interaction with the water molecule, leading to a depressed drop shape and low contact angle. In the reverse, low surface energy leads to low interaction with the water molecule, leading to a spherical drop and high contact angle [23]. As shown in Figure 1, the ceramic mixture composition strongly affects the wettability. The wettability of zirconia containing composites is, in fact, higher than pure titania–alumina ones. The high wettability of the sintered zirconia was already observed by Yilbas et al. (θZrO_2_/H_2_O = 51°) [24]. Therefore, the contact angle between water and pure zirconia can be lower than alumina one [25]. Furthermore, a significant increase of the contact angle of zirconia-based ceramics was observed when adding 5 wt.% and 10 wt.% TiO_2_. This phenomenon has been attributed to the wettability difference between the zirconia and titania, the former being more hydrophilic than the latter [26,27]. This influences the water interaction with the ternary composite ceramic, inducing a progressive increase of the contact angle, increasing titania and alumina oxides. 

On the other hand, concerning alumina rich mixtures, the addition of different amounts of titania to alumina induces the decrease of the contact angle. Zhang et al. showed that the contact angle of water on alumina is higher than on the titania surface, which confirm our results [28]. 

In addition, the presence of oxygen vacancies in titania–alumina ceramics may lead to enhanced wettability. The sintered titania doped alumina is, in fact, characterized by the presence of oxygen vacancies which are a result of the presence of aluminums in rutile lattice as a defect with a single negative charge AlTi’ according to the following reaction:

(1) $$Al_{2}O_{3}\to 2Al_{Ti^{’}}+V\ddot{o}+3O_{o}^{x}$$

Increasing the titania content, these vacancies increase [13]. As shown by Samandi et al. the formation of defects such as vacancies and interstitials would result in decreasing the contact angle (enhanced wettability) [29].

In Alumina–Zirconia ceramics, the addition of titania does not induce any significant effect on the wettability of composites which is probably due to the formation of intermediate phases such as A_l2_TiO_5_ and ZrTiO_4_. In our previous study [13], we proved, in fact, that the addition of TiO_2_ in zirconia and alumina stimulated the formation of intermediate phases due to its high reactivity.

The contact angle values of studied ceramic composites ranged from 53° to 81°, indicating that, notwithstanding the variability, the wetting liquid was being drawn towards the surface. These contact angles, lower than 90°, designate surfaces as hydrophilic and thus they display high wettability of ceramic composites. The hydrophilic ceramic surfaces can facilitate the physiological interactions between body fluid and implant to obtain a suitable cell adhesion and, consequently, an optimal osseointegration [30,31,32]. According to Anselme et al. [33], the wettability of alumina and zirconia is higher than that of metals and polymers used for orthopedic application thus promoting the osseointegration of the implant. In addition, Hao et al. found better protein [34] human fibroblast [35] and osteoblast [36] attachment on the magnesia partially stabilized zirconia when it was characterized by higher wettability characteristics.

### 3.2. Cytotoxicity

#### 3.2.1. Cell Viability

**Figure 2:**
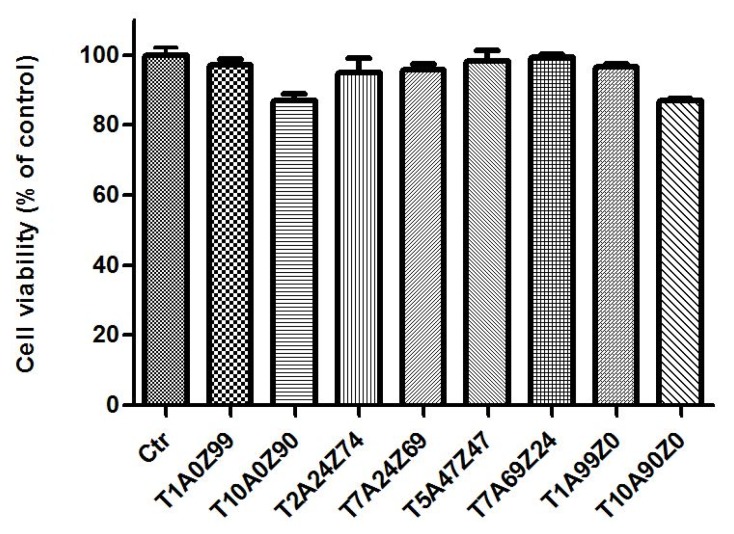
Cell viability of human gingival fibroblast (HGF-1) cells cultured on the composite samples for three days.

Other aspects to be taken into consideration to characterize new biomaterials are cytotoxicity and genotoxicity. The cell viability of HGF-1 cells cultured for three days over the composite samples is presented on Figure 2. The values are similar in the five compositions containing less than 10 wt.% TiO_2_. The cell viability is higher than 95%. Therefore, it can be assumed that the variation of Al_2_O_3_ and ZrO_2_ content did not induce significant changes in the cell viability. These results were confirmed by Sequeira et al. showing similar cell viability and proliferation in both ATZ (Alumina Toughened Zirconia) and ZTA (Zirconia Toughened Alumina) composites [37]. In addition, according to Marchi et al. [38], the mixing of ceramics that have differences in bioactivity could lead to a similar cell proliferation. However, T10A0Z90 and T10A90Z0 composites, containing 10 wt.% of TiO_2_, presented lower values compared to other samples. This was previously confirmed by the study carried out by Agac et al., which reported that the co-doping of zirconia with titanium dioxide and alumina induced higher cell viability and attachment than the doping of zirconia with only titanium dioxide [12]. A high value of cell viability was expected on the mixtures containing the highest amount of titania because of its well-known bioactivity. However, it was observed that the presence of titania containing secondary phases could significantly alter such behavior. Furthermore, as shown by Kaluđerović et al., the proliferation of cells cultured on zirconia containing 11–13% TiO_2_ was significantly lower compared to that on pure zirconia [39], thus confirming that the chemical compositions and secondary phase’s presence and distribution are important determinants of cell behavior [38]. 

However, all composites showed a cell mortality that did not exceed 17%. Therefore, according to the international standard guide ISO 10993-5, that classifies a cytotoxic effect as a decrease in cell viability by more than 30%, we can confirm that our studied composites are not cytotoxic [40,41]. 

#### 3.2.2. Relationship between Cell Viability and Surface Wettability

**Figure 3:**
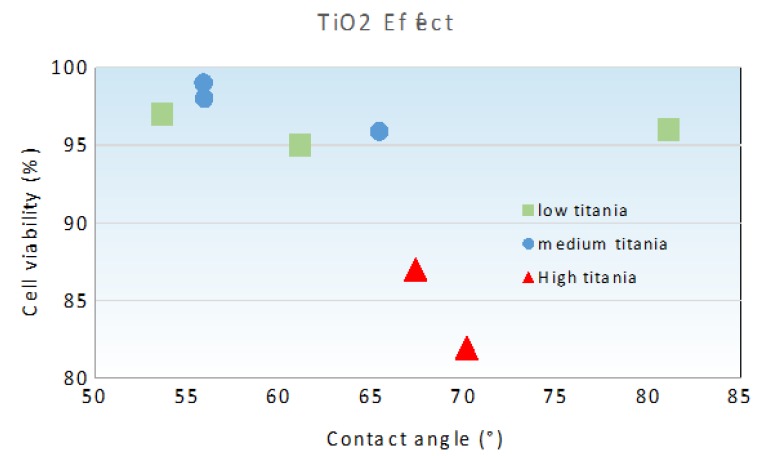
Variation of cell viability with contact angle of investigated ceramics.

In order to understand the relationship between wettability and cell viability, the effect of the contact angle of different ceramic composites on their cell viability is shown in Figure 3. We can distinguish three families. The first family includes low titania content ceramics: T1A0Z99, T1A99Z0, and T2A24Z74. The second family consists of medium titania content ceramic composites (T5A47Z47, T7A24Z69, and T7A69Z24), while high titania content ceramics (T10A0Z90 and T10A90Z0) represent a third distinct ceramic family.

The cell viability of human gingival cells on low and medium titania content composites is high, thus it is not significantly influenced by changes in wettability behavior. This aspect is more notable in low titania content ceramics with a contact angle as high as 81° which, however, still showed a cell viability higher than 95%. Moreover, the effect of wettability on cell proliferation was improved by adding 5 wt.% of TiO_2_. This result was expected because the titania is known by its high bioactivity [42]. The viability/wettability relationship for the high titania content family is the most important. Indeed, the effect of wettability on cell viability is more pronounced when the titania content increases from 5 wt.% (medium titania family) to 10 wt.% (high titania family). This effect is likely due to the formation of secondary phases. The titanium dioxide solubility in alumina and zirconia is limited, in fact. Above this solubility value, a further addition of TiO_2_ induced the formation of secondary phases, such as zirconia–titania and alumina–titania. According to Capel et al., stable titania–zirconia solid phases could be obtained in these conditions [43].

As shown in our previous study [13], the oxides’ interactions are more important in a high titanium dioxide system than in a medium titania one, thus the content of secondary phases is higher in high TiO_2_ ceramic composites which have a crucial role on wettability and, consequently, on cell viability. 

Moreover, for different titania families, the cell viability increases when the wettability increases, corresponding to the decrease of contact angle. These results can be explained by observing the adsorbed water content on the ceramic surface that can enhance the cell proliferation, i.e., human cells are more likely to adhere to a wet surface. Furthermore, Marchi et al., in their study of the biocompatibility of mixed ceramic composites using human gingival fibroblast cell lines, showed that H_2_O functional groups revealed by the DRIFTS (Diffuse Reflectance Infrared Fourier Transform) analysis on surface of the ceramics control the cell adherence and growth [38].

#### 3.2.3. Cell Morphology

**Figure 4:**
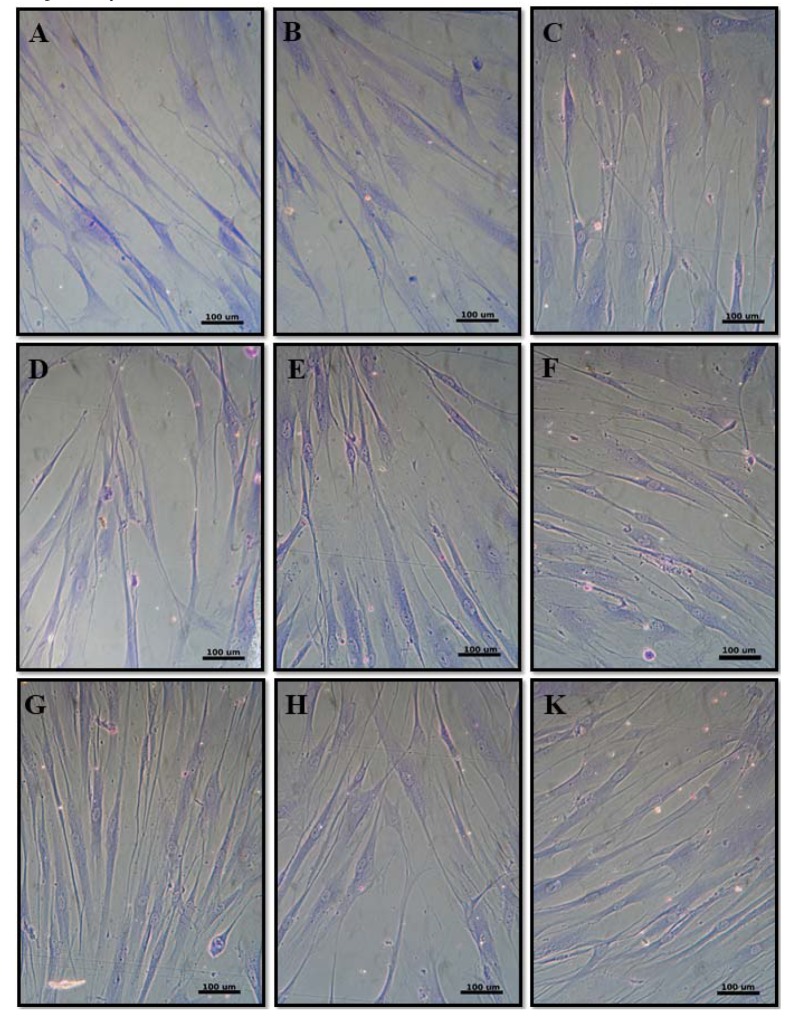
Cell morphology of negative control (**A**), T1A0Z99 (**B**), T10A0Z90 (**C**), T1A99Z0 (**D**), T10A90Z0 (**E**), T5A47Z47 (**F**), T2A24Z74 (**G**), T7A24Z69 (**H**), and T7A69Z24 (**K**).

Human gingival fibroblast cells showed a typical spindle-shaped morphology with extended cellular processes like filopodi and lamellopodia [44]. No marked morphological changes were observed between the control and fibroblasts cultured with different composites (Figure 4). The absence of cytotoxicity was also indicated by the nonappearance of morphological alterations in HGF-1 cells such as a loss of spindle shape and cell lysis. This result was in line with previous studies on cells in culture with alumina and zirconia ceramics [45]. 

However, these aspects, which are of fundamental importance in the proper selection of bioceramic materials, need to be investigated further.

According to published guidelines [46], the determination of genotoxic potential of materials must follow the cell viability study because DNA damage can be an important confounding factor. Hence, we studied the genotoxicity of our composites via the comet test in order to confirm their biocompatibility.

### 3.3. Genotoxicity

**Figure 5:**
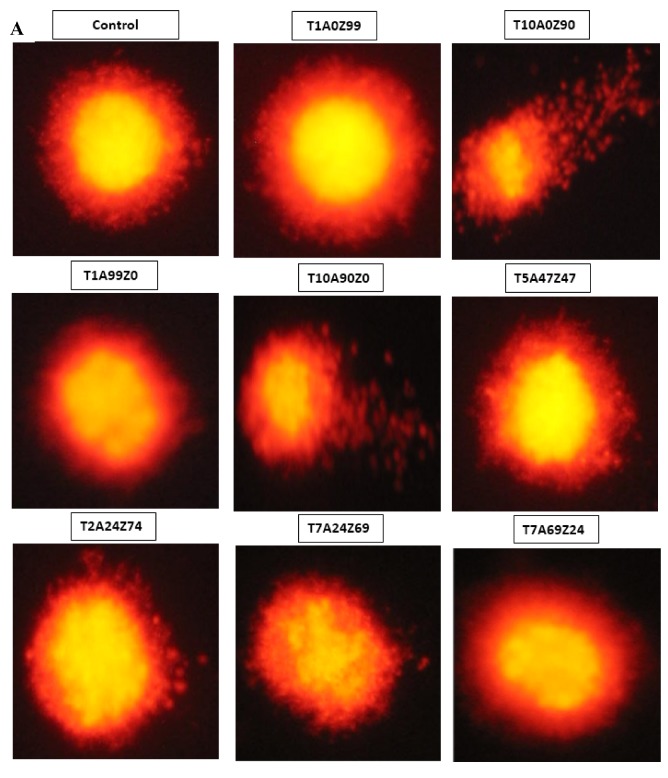

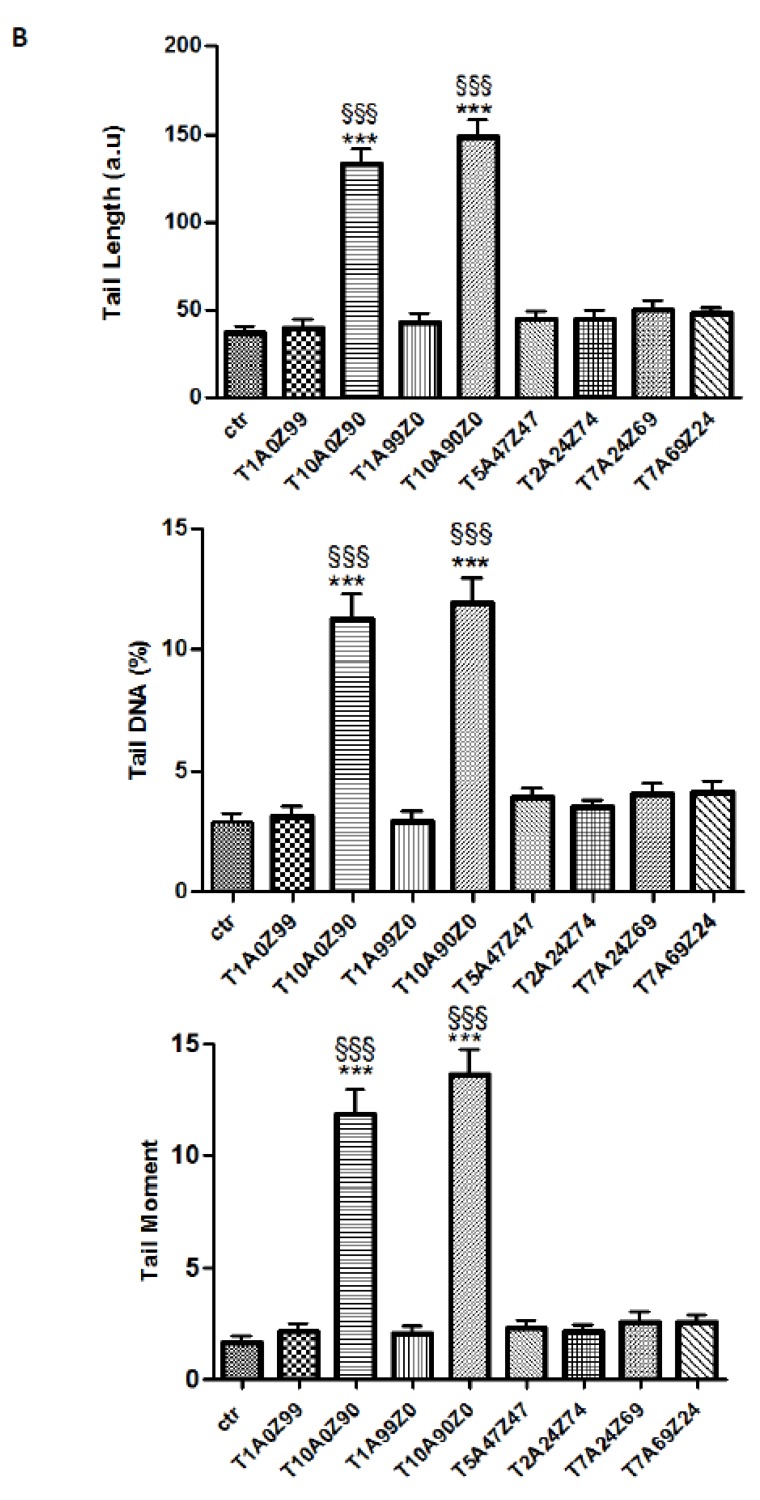
DNA damage analysis of differentiated HGF-1 cells. (**A**) Representative images of the comet assay performed in the presence or absence of ceramic composites. (**B**) DNA damage was evaluated by measurement of tail length (TL), Tail DNA (%) and tail moment (TM) in CASP software. Values are shown as mean ± standard error of the mean (SEM). *** *p* < 0.001 in comparison with controls; §§§ *p* < 0.001 in comparison with cells cultured with different composites.

Typical SCGE images for the control and each composite were shown in Figure 5A. SCGE images were captured and analyzed by the CASP package, and the tail length, tail DNA%, and tail moment of all the 100 selected cells of each experiment group was calculated (Figure 5B). According to tail length results, T10A0Z90 (133.09 ± 8.66) and T10A90Z0 (148.51 ± 10.08) showed significant differences with negative control (36.86 ± 3.56) and with other composites (T1A0Z99, T1A99Z0, T5A47Z47, T2A24Z74, T7A24Z69, and T7A69Z24) (*p* < 0.001). Similar results were obtained in the tail DNA% and tail moment analysis. None of the T1A0Z99 (3.08 ± 0.47), T1A99Z0 (2.91 ± 0.46), T5A47Z47 (3.92 ± 0.40), T2A24Z74 (3.48 ± 0.29), T7A24Z69 (4.05 ± 0.46), and T7A69Z24 (4.09 ± 0.53) cell groups showed statistically significant differences with the negative control (2.90 ± 0.36) (*p* > 0.001), in fact, while T10A0Z90 (11.24 ± 1.08) and T10A90Z0 (11.89 ± 1.05) were significantly different with these composites and negative controls (*p* < 0.001). As indicated by tail moment results, none of the T1A0Z99 (2.12 ± 0.39), T1A99Z0 (2.03 ± 0.37), T5A47Z47 (2.28 ± 0.39), T2A24Z74 (2.16 ± 0.28), T7A24Z69 (2.57 ± 0.49), and T7A69Z24 (2.56 ± 0.35) cell groups showed differences with negative control (1.67 ± 0.25) (*p* > 0.001), while T10A0Z90 (11.85 ± 1.07) and T10A90Z0 (13.61 ± 1.14) were significantly different with these composites and negative control (*p* < 0.001). 

These results lead to the conclusion that the composites containing 10 wt.% TiO_2_ induced DNA damage. There was, indeed, a significant difference in the tail length, the tail DNA%, and the tail moment between the negative control and the two composites T10A0Z90 and T10A0Z90. On the other hand, the composites that had a lower percentage of TiO_2_ (0.27; 2.5; 5; 7.5 wt.%) did not induce any significant DNA damage. 

Previous studies highlighted that zirconia and alumina ceramic materials do not show any genotoxicity risk, based on in vitro tests [47,48]. 

These biocompatibility capabilities are preserved also on binary zirconia–alumina composites, as assessed by Maccauro et al. [49]. Their results, based on DNA damage, mutagenicity, and cancerogenetic potential in mammalian cells, confirmed that ZTA material could be considered suitable for biomedical applications. The addition of titania could induce non-significant genotoxic effects [50,51,52]. However, as observed by Lu et al. [53], who studied the effects of titania on the induction of sister chromatid exchanges and micronuclei in Chinese hamster ovary-K1 cells, the titania could have a potentially genotoxic effect. This detrimental action could be due to the high dose of TiO_2_ that could stimulate the DNA damage, thus limiting the threshold TiO_2_ percentage on ceramic composites for biomedical applications [54,55].

By comparing the tail length, tail DNA%, and the tail moment of different composites among themselves and with the control, we noted that the two composites T10A0Z90 and T10A90Z0, which contain the highest content of titania (10 wt.%), are statistically different from other ceramic composites and control. These results were previously confirmed by cell viability results. Indeed, the minimum values of cell viability were reached with these two composites. Thus, the genotoxic effect only appears with composites containing 10 wt% TiO_2_, which is probably due to titania content, previously confirmed to have a great influence on cell viability, wettability, and ceramic phase content. As shown by Marchi et al., the presence of secondary phases is an important determinant of the DNA-damage of materials [38]. 

To summarize, the aim of the present work was to evaluate if different composites in the alumina–zirconia–titania system are able to integrate with human host tissues by studying their wettability, cytotoxicity, and genotoxicity in vitro. In vitro tests have gained large acceptance among biocompatibility tests because the methodology is able isolate potential biases from the environment and assesses subtle cell metabolism modifications [56], allowing for the evaluation of data with higher accuracy [57]. The results revealed that the wettability was adequate enough to have potentially reliable osseointegration behaviors for dental implantology. Indeed, the ceramic composites can promote cell adhesion and bonding in the tissue and implant interface, and thus minimize the clinical failure rates of implants.

Regarding cytotoxicity, all the tested composites did not cause significant cell death, according to the ISO 10993 standard. In addition, it is assumed that the genotoxicity to human gingival fibroblasts induced by different ceramic composites was dose-dependent.

Considering that the in vitro tests do not take into account the complex homeostatic phenomenon that takes place in the human body, clinical studies in vivo are desirable in order to further assess the biological behavior of these dental ceramic composites on human cells, and to study the osseointegration when they are used as dental implant material, along with the osteoinductive activity when they are used as bone grafting implants.

## 4. Conclusions

The ceramic composites presented in this study were originally developed for the improvement of clinically used biomaterials. Several compositions were tested and wettability behavior, cytotoxicity, and genotoxicity were studied. The results are summarized as follows.

- The studied ceramic composites with high wettability could be used to manufacture dental implants with a high capacity of osseointegration.
- The results of the biological tests highlighted the absence of cytotoxicity when compared to the control.
- The DNA damage is closely related to TiO_2_ content. Genotoxicity towards human gingival fibroblasts was mainly attributed to composites containing 10 wt.% TiO_2_.

## Figures and Tables

**Table 1:** Codes and details of components amount (wt.%).

Code	TiO_2_	Al_2_O_3_	ZrO_2_ (12 mol.% CeO_2_)
T1A0Z99	0.27	0	99.73
T10A0Z90	10	0	90
T1A99Z0	0.27	99.73	0
T10A90Z0	10	90	0
T5A0Z95	5	0	95
T1A50Z50	0.27	49.87	49.87
T10A45Z45	10	45	45
T5A95Z0	5	95	0
T5A47Z47	5	47.50	47.50
T2A24Z74	2.5	23.75	73.75
T7A24Z69	7.5	23.75	68.75
T2A74Z24	2.5	73.75	23.75
T7A69Z24	7.5	68.75	23.75
